# Death Literacy and Death Competence in Undergraduate Clinical and Allied Health Education: Protocol for a Mixed Methods Study

**DOI:** 10.2196/86867

**Published:** 2026-01-19

**Authors:** Sora Lee, Kerrie Noonan, Andrea Grindrod, Sumina Shrestha, Niki Read

**Affiliations:** 1 La Trobe University Bundoora, Victoria Australia

**Keywords:** death literacy, death competency, end-of-life care, health care education, palliative care, curriculum development

## Abstract

**Background:**

End-of-life care is a critical competency for the health care workforce, yet evidence suggests that many health care professionals feel unprepared to engage with death, dying, and bereavement. Death literacy and death competence are emerging frameworks for assessing readiness to provide high-quality, compassionate care. Although validated tools exist, little is known about the preparedness of final-year undergraduate health care students in Australia. Understanding their current levels of death literacy and death competence is essential for informing curriculum design and strengthening workforce capacity.

**Objective:**

This study aims to (1) measure death literacy and death competence among final-year students in medicine, nursing, and allied health programs in Australian universities; (2) explore students’ reflections on how undergraduate training has shaped their preparedness for end-of-life care; and (3) identify educational needs and opportunities for curriculum enhancement.

**Methods:**

A mixed methods design will be used. An online survey (15-20 minutes) will be distributed to final-year students across multiple Australian universities. The survey includes the Death Literacy Index, the Death Competency Scale, and open-ended reflection questions. Quantitative data will be analyzed using descriptive and inferential statistics (in SPSS and Stata), with subgroup comparisons across disciplines and benchmarking against national professional datasets. Qualitative responses will be analyzed thematically. In phase 2, up to 20 students will participate in 2 focus groups (60-90 minutes each). The focus groups will explore survey findings and students’ perceptions of training, preparedness, and gaps. Data will be transcribed, anonymized, and analyzed thematically using NVivo.

**Results:**

Data collection for the national survey is scheduled from September 2025 to December 2025, with an anticipated sample of 60 to 120 final-year students across medicine, nursing, and allied health disciplines. Data analysis will begin in March 2026, and findings are expected to be published in late 2026. The findings will establish baseline measures of death literacy and death competence among final-year health care students and identify strengths and gaps in current curricula. Results will be synthesized to provide actionable insights for educators and to inform future intervention studies.

**Conclusions:**

By providing the first Australian pilot data on death literacy and death competence among final-year health care students, this study will inform curriculum development and workforce planning. The findings have the potential to enhance educational strategies, improve the preparedness of graduates for delivering end-of-life care, and contribute to the development of a death-literate health system.

**International Registered Report Identifier (IRRID):**

DERR1-10.2196/86867

## Introduction

### Background

Population aging and the rising prevalence of chronic, life-limiting illnesses mean that most health care professionals will inevitably care for patients at the end of life. Delivering high-quality, compassionate care in these circumstances requires not only clinical competence but also effective communication, psychosocial support, and emotional resilience. However, many medical and nursing graduates report feeling underprepared to manage death and dying in practice [1]. Therefore, the preparedness of the future workforce is a pressing concern for both health care education and health system planning.

Two complementary frameworks have become increasingly important in assessing readiness for death-related work: death literacy and death competence. Death literacy refers to the knowledge, skills, and experiential learning that enable individuals and communities to understand, navigate, and engage with death, dying, and bereavement [2]. It incorporates both practical and social dimensions, including the ability to draw on networks of support and to foster open discussion about death [3]. Death literacy is emerging as a valuable concept for professionals and the wider community, contributing to more compassionate and participatory approaches to end-of-life care.

Death competence, while related, emphasizes the personal and professional capacities required to care effectively for dying patients. These capacities include self-efficacy, communication skills, the ability to cope with emotional demands, and the ability to sustain compassionate care under pressure [4]. Higher levels of death competence have been linked to greater practitioner confidence, reduced death anxiety, and enhanced quality of care [5-8]. Validated tools such as the Death Competency Scale (DCS) [4,9] provide structured means of assessing these capacities among both practicing professionals and trainees.

Although these constructs are increasingly studied internationally, a clear gap remains in the Australian context regarding undergraduate health care education. Existing research has typically examined practicing professionals or early graduates, often highlighting deficiencies in end-of-life preparedness and recommending greater emphasis on structured education and experiential learning [1,10]. Furthermore, while there has been one published study on death literacy among psychology students [11], research on the death literacy and death competence of students at the point of graduation is lacking in an Australian context. Understanding the baseline levels of preparedness among final-year students is crucial for informing curriculum development, aligning educational strategies with workforce needs, and ultimately improving the delivery of end-of-life care.

### International Perspectives

Research from the United Kingdom shows that many medical students and junior doctors feel poorly prepared to care for dying patients [12]. Surveys consistently demonstrate low levels of confidence and high levels of distress among graduates, despite the inclusion of palliative care in undergraduate curricula [13]. A cross-institutional review of UK medical schools found substantial variability in palliative care teaching, with weekly teaching hours ranging from less than 1 to more than 16 [14]. Similarly, 88% of US medical residents reported that they had little or no training during residency [15].

In European contexts, particularly Germany and the Netherlands, studies have highlighted inconsistent integration of palliative care into undergraduate education. Although awareness of the importance of end-of-life training is growing, student exposure to real clinical encounters with dying patients remains uneven [14]. Research with nursing students in Turkey suggests that high levels of spirituality may influence death literacy, hence recommending that nursing students have access to a curriculum that encourages exploration of death literacy and spirituality [16]. Nursing curricula that specifically provide both didactic and exploratory instructions along with practical experience have also been shown to improve death competence and death literacy [17].

In Asia, cultural taboos surrounding death further complicate training. In Hong Kong, for example, students often struggle with death-related conversations, influenced both by societal norms and by insufficient curriculum space for end-of-life care [18]. Similarly, research from Taiwan and mainland China suggests that health and social care students experience heightened anxiety and reduced confidence when discussing death, reinforcing the importance of context-specific training approaches [10,19].

North America has a strong tradition of conducting death education programs in undergraduate health and medical training [20,21]. Simulation-based training has been adopted to enhance student preparedness. A national study demonstrated that simulated experiences with dying patients improved both confidence and communication skills among medical trainees [22]. Despite these innovations, gaps persist in graduates’ ability to manage the emotional and ethical complexities of caring for people who are dying and their families.

Together, these international studies highlight a common pattern: although death literacy and death competence are increasingly recognized as essential, systematic assessments of student preparedness at the point of graduation remain limited. Comparative data also suggest that curriculum alone is insufficient unless combined with experiential learning that is meaningful and structured and provides an opportunity for personal reflection.

### Rationale and Study Aims

Despite international advances in death literacy, there are no published studies examining the skills, knowledge, and death competence among medical, nursing, and allied health students in Australia. Addressing this gap is important for three reasons: (1) educational insight to establish baseline measures of knowledge, skills, and preparedness for end-of-life care among graduating students, benchmarked against practicing professionals and the general population; (2) curriculum development to identify strengths and gaps in existing training programs, as perceived by students, and to generate evidence for guiding curriculum reform; and (3) workforce readiness to provide actionable information on how well prepared the future workforce is to deliver compassionate and competent end-of-life care, thereby strengthening Australia’s death-literate health system.

Accordingly, this study aims to accomplish the following objectives:

1. Examine the levels of death literacy and death competence among final-year undergraduate health care students
2. Explore qualitatively how students reflect on their training, preparedness, and perceived gaps in education related to death and dying
3. Generate recommendations for enhancing health care professional curricula to better prepare the future workforce for end-of-life care

By combining validated survey instruments with focus group discussions, this mixed methods protocol will generate both benchmarking data and rich insights into students’ lived educational experiences. Together, these findings will provide a foundation for targeted curriculum innovations and policy strategies to build a death-literate and death-competent health care workforce in Australia.

### Benchmarking of Death Literacy Index Scores

The proposed benchmarking against national professional datasets is intended as a *contextual comparison* and not a statistical equivalence test. Benchmark data will be drawn from published aggregate results in the Australian National Death Literacy Index survey (2024), which includes a subset of practicing clinicians and related professional practice studies [23].

As the Australian benchmark data were collected using the same validated Death Literacy Index (DLI) under similar online conditions, mean score comparisons will be descriptive in nature. These comparisons will be accompanied by effect-size estimates and narrative interpretation rather than formal significance testing.

## Methods

### Study Design

This study uses a mixed methods design combining a cross-sectional online survey (15-20 minutes) with qualitative focus group discussions (60-90 minutes each). The survey component will establish baseline measures of death literacy and death competence among final-year health care students, whereas the qualitative component will provide depth and contextual understanding of students’ experiences and perceptions of preparedness for end-of-life care. Insights from both components will be analyzed to inform and strengthen curriculum development in this area.

### Participants

Eligible participants are final-year undergraduate students enrolled in medicine, nursing, and allied health programs (including psychology, paramedicine, midwifery, and social work) across Australian universities. The following 9 universities have agreed to support recruitment: the University of Sydney, Western Sydney University, Charles Sturt University, the University of Newcastle, La Trobe University, Swinburne University, Victoria University, Monash University, and the University of Melbourne. The study population, sample size, and eligibility criteria are outlined as follows:

1. Survey sample size of 60-120 respondents (pilot estimate, with a 10%-20% expected response rate)
2. Focus group sample size of up to 20 participants, divided into 2 groups of 8 to 10 each, providing sufficient qualitative data for thematic analysis
3. Inclusion criteria are final-year enrollment in one of the target programs, age ≥18 years, and consent to participate
4. Exclusion criteria are enrollment in earlier years of study or outside the specified institutions

### Sample Size and Analytic Scope

The initial recruitment target of 60 to 120 final-year students is appropriate for a pilot study designed to estimate variability and assess feasibility rather than to conduct fully powered inferential analyses. We acknowledge that subgroup analyses (eg, medicine vs nursing and allied health; metropolitan vs rural) may be underpowered and will therefore be treated as exploratory and descriptive rather than confirmatory. Results will focus on precision and CI estimates, with formal hypothesis testing deferred to subsequent, larger studies once effect-size estimates are available. This approach is consistent with standard pilot study methodology and the emphasis on proportionality of design to purpose in the 2023 National Statement on Ethical Conduct in Human Research [24].

### Recruitment

Recruitment will occur through professional networks and university course coordinators who will circulate email invitations and postlearning management system announcements.

For survey recruitment, students will receive a link to the online questionnaire via QuestionPro, version 2024 (QuestionPro Inc). Participation will be anonymous and will not identify individual students or universities. Consent will be obtained electronically before participation, with students indicating their agreement by selecting the option to begin the survey.

For focus group recruitment, at the end of the survey, students will be invited to complete a separate expression-of-interest form. Those selected (based on program representation and availability) will be contacted by the research team, provided with participant information and consent forms, and scheduled for either an in-person or online session.

Course coordinators will not be informed of which students choose to participate, and researchers will have no direct teaching or assessment responsibilities for the eligible student cohorts.

### Instrument

The online survey will include (1) demographics, including age, gender, program of study, university, and previous experience with death and dying; () the DLI a validated 29-item measure assessing knowledge, skills, and experiential learning related to death and dying [23]; (3) the Bugen Death Competence Scale (Coping with Death Scale), short version, a validated 9-item questionnaire measuring self-efficacy, emotional coping, and preparedness for death work based on the original 30-item scale [4,10,25]; and (4) open-ended questions, comprising items to reflect on students’ training, the perceived strengths and gaps, and suggestions for curricular improvements.

Focus groups will follow a semistructured guide, exploring reactions to descriptive survey findings, perceptions of preparedness for end-of-life care, experiences of training that were most or least helpful, and recommendations for educational improvements. Sessions will be facilitated by experienced palliative care researchers, audio recorded (with consent), and transcribed verbatim.

### Data Analysis

#### Quantitative Data

Descriptive and inferential analyses will be conducted using SPSS (version 29.0; IBM Corp) and Stata (version 18.0; StataCorp LLC). Scaled mean scores on DLI, and mean scores on the DCS will be calculated, and subgroup analyses will compare results across disciplines (medicine, nursing, and allied health) and geographic location (metropolitan and rural). Results will be benchmarked against national DLI data from practicing professionals.

#### Qualitative Data

Open-ended survey responses and focus group transcripts will be analyzed thematically following the 6-step framework by Braun and Clarke [25]. NVivo (version 14; Lumivero) will be used to support coding and theme development. Analysis meetings will document coding decisions to ensure reflexivity and consistency.

#### Integration

A mixed methods synthesis will compare quantitative benchmarks with qualitative themes, identifying areas of convergence (eg, perceived strengths) and divergence (eg, unmeasured concerns about training). Illustrative quotations will be included in the reporting to enrich interpretation.

### Ethical Considerations

This study has received ethics approval from the La Trobe University Human Research Ethics Committee (HEC 25370). All participants will provide informed consent before participation. For the online survey, consent will be indicated electronically via an information and consent page. For focus groups, written consent will be obtained before participation, with verbal consent reconfirmed at the beginning of each session. While there is no remuneration for survey participants, each focus group participant will be compensated Aus $50 (US $32.50). Participation is voluntary, and students may withdraw at any time before survey submission or at any point before or during focus group discussions. All data will be deidentified during transcription, and confidentiality will be maintained through secure storage on password-protected university servers. Participants will also be provided with information about available support services, given the sensitive nature of discussions around death and dying.

### Timeline

As shown in Table 1, the timeline for the project spans October 2025 to February 2026, encompassing survey data collection (October 2025 to December 2025), qualitative data collection (November 2025 to February 2026), analysis (March 2026 to September 2026), and dissemination (October 2026 to December 2026).

**Table 1:** Study timeline and milestones.

Timeline, year (quarter)	Activity and milestones	Output and deliverables
2025 (Q3)	Ethics approval obtained	Approved study protocol
2025 (Q3)	Survey preparation (September)	Online survey finalized
2025 (Q4)	Survey data collection (October-December)	Online survey responses collected
2025 (Q4) to 2026 (Q1)	Focus group data collection (November 2025-February 2026)	Audio recordings and transcripts
2026 (Q1)	Quantitative data analysis (January-March)	Descriptive or comparative analysis
2026 (Q1)	Qualitative data analysis (March-June)	Thematic coding and analytic memos
2026 (Q2)	Integration of findings and manuscript preparation	Draft manuscripts for submission
2026 (Q3)	Dissemination	Journal submission and conference abstracts

## Results

### Study Timeline

Funding for this project was awarded in August 2025. Recruitment and data collection for the online survey are planned for October 2025 to December 2025, with a target sample of 60 to 120 final-year students enrolled in Australian medicine, nursing, or allied health programs. Focus group recruitment will follow immediately after the survey closes, with sessions anticipated for November 2025 and February 2026. Data cleaning and analysis will commence in March 2026, and dissemination of findings is planned for late 2026.

### Quantitative Outcomes

The survey will generate descriptive statistics on death literacy and death competence levels across medicine, nursing, and allied health disciplines. Subgroup analyses are anticipated to reveal variation by discipline (eg, higher preparedness among nursing students relative to other groups, consistent with international findings) and by geographic location (ie, metropolitan and rural). An exploratory study on benchmarking against existing professional population data from the DLI will provide insight into how well undergraduate training prepares students compared to practicing clinicians.

### Qualitative Outcomes

Open-ended survey responses and focus group discussions are expected to provide rich insights into student perceptions of preparedness. Anticipated themes include strengths of current training (eg, clinical placements and communication skills workshops), gaps or barriers in curricula (eg, limited experiential exposure to dying patients and lack of reflective spaces), emotional and cultural challenges in engaging with death and dying, and recommendations generated by students for curriculum enhancement.

### Integrated Insights

By combining quantitative benchmarks with qualitative reflections, the study will identify both measurable outcomes and contextualized experiences. This mixed methods integration is expected to highlight areas where curricula can be further development to prepare graduates for working with people with life-limiting illnesses.

### Dissemination Plan

Findings will be disseminated through peer-reviewed journal publications and conference presentations in palliative care, health education, and public health forums. A summary of results will also be shared with participating universities to inform curriculum development and training strategies.

### Long-Term Impact

Although exploratory in scope, this study will provide a baseline dataset that can inform larger-scale studies and intervention trials. Ultimately, the anticipated outcomes will contribute to building a death-literate health care workforce, better equipped to support patients, families, and communities in end-of-life contexts.

## Discussion

### Anticipated Findings

This study is expected to show meaningful variation in death literacy and death competency levels among final-year medicine, nursing, and allied health students. We anticipate that students with greater exposure to palliative or end-of-life education will demonstrate higher DLI and DCS scores and that gaps will emerge between discipline groups. These anticipated findings will help identify priority areas for curriculum strengthening and workforce readiness.

This pilot study addresses a clear evidence gap by assessing death literacy and death competence among final-year health care students in Australia. International research has consistently shown that many students feel ill-equipped to care for dying patients [13,14]; however, no comparable Australian data examining death literacy currently exists. By situating student preparedness within the frameworks of death literacy and death competence, this study positions end-of-life education as both a professional requirement and a public health priority.

Based on previous national surveys and training evaluation research, we expect to observe moderate overall levels of death literacy, with notable gaps in system navigation, communication readiness, and community engagement components. Differences between disciplines and training environments are also anticipated. Previous Australian DLI population studies [26] indicate wide variability in death literacy across the general population. Existing research with early-career clinicians highlights insufficient preparation for end-of-life communication and care planning. This study aims to extend that evidence by providing pregraduation baselines among future clinicians, which has not yet been mapped at scale.

The findings will provide actionable evidence for supplementary education on end-of-life care in medical, nursing, and allied health programs. They will help educators identify which aspects of training best support preparedness and where additional strategies—such as simulation, reflective practice, and structured communication skill building—may be needed [22]. Beyond the classroom, stronger graduate readiness is likely to improve the quality of communication and care for patients and families and to contribute to building death-literate health systems that normalize end-of-life discussions.

### Strengths

A key strength of this project is its mixed methods approach, combining validated survey measures with qualitative focus groups to provide both breadth and depth. The multi-institutional recruitment strategy enhances the diversity of student perspectives, while the involvement of an interdisciplinary research team ensures methodological rigor.

### Limitations

As a pilot, the study is not designed for large-scale generalization. The modest sample size may limit the precision of subgroup comparisons, and voluntary participation may introduce self-selection bias. Focus groups are restricted to 2 sessions, which may not capture the full spectrum of student experiences. Furthermore, given the sensitivity of the topic, some participants may choose not to disclose personal reflections. Other limitations include possible underrepresentation of certain disciplines and variability in exposure to death and dying curricula.

### Future Directions

Despite these limitations, the study will establish a baseline for future, larger-scale research and intervention trials. The mixed methods framework can be expanded nationally to track changes in student preparedness over time and across institutions. Longer term, the findings may inform accreditation standards and professional development frameworks, embedding death literacy and death competence more systematically into health care education.

### Conclusions

This study will provide the first national snapshot of death literacy and death competence levels among final-year Australian health care students. By establishing baseline measures and identifying educational gaps, the project will lay the groundwork for evidence-informed curriculum design and targeted end-of-life training for the future health workforce.

## Abbreviations

DCS: Death Competency Scale
DLI: Death Literacy Index

## Data Availability

Data generated in this study will not be publicly available due to confidentiality requirements.

## References

[1] Deravin L, Karin Anderson J, Croxon L (2016). Are newly graduated nurses ready to deal with death and dying? - a literature review. Nurs Palliat Care.

[2] Noonan K, Horsfall D, Leonard R, Rosenberg J (2016). Developing death literacy. Prog Palliat Care.

[3] Graham-Wisener L, Toner P, Leonard R, Groarke JM (2022). Psychometric validation of the death literacy index and benchmarking of death literacy level in a representative UK population sample. BMC Palliat Care.

[4] Robbins RA, Neimeyer RA (1994). Death competency: Bugen's coping with death scale and death self-efficacy. Death Anxiety Handbook: Research, Instrumentation, And Application.

[5] Cheung JT, Au DW, Chan WC, Chan JH, Ng K, Woo J (2018). Self-competence in death work among health and social care workers: a region-wide survey in Hong Kong. BMC Palliat Care.

[6] An R, Zhang SF, Huang XX, Zhao XY, Cao T, Bai L, Wan QQ (2022). Self-reported practices, competence and difficulties towards palliative care among nurses: a cross-sectional study. Eur J Cancer Care (Engl).

[7] Wang Y, Huang Y, Zheng R, Xu J, Zhang L, Zhu P, Lu Z, Wang L, Xie J, Zhao J, Dong F (2023). The contribution of perceived death competence in determining the professional quality of life of novice oncology nurses: a multicentre study. Eur J Oncol Nurs.

[8] Guo Q, Wang Y, Zheng R, Wang J, Zhu P, Wang L, Dong F (2024). Death competence profiles and influencing factors among novice oncology nurses: a latent profile analysis. BMC Nurs.

[9] Bugen LA (1981). Coping: effects of death education. Omega.

[10] Lin Z, Lou VW, Chan WC (2025). Validating the self-competence in death work scale for end-of-life care volunteers. BMC Palliat Care.

[11] Vivekananda K, Parratt C, Tucker M, Leonard R (2021). The impact of online death literacy education on psychology students to have better end‐of‐life conversations. Aus Psychol.

[12] Moloney C, Nuzum D, Lyons M, Steele C, Forrest C, Lynch E, Elhassan E, O'Sullivan H, O'Leary MJ, O'Reilly S (2025). Dying, death and doctors-in-training: survey. BMJ Support Palliat Care.

[13] Gibbins J, McCoubrie R, Forbes K (2011). Why are newly qualified doctors unprepared to care for patients at the end of life?. Med Educ.

[14] Weber M, Schmiedel S, Nauck F, Alt-Epping B (2011). Knowledge and attitude of final - year medical students in Germany towards palliative care - an interinstitutional questionnaire-based study. BMC Palliat Care.

[15] Patterson L, Decker A, King A, Roman A, Bolkan C, Weaver RH (2025). Preparing for the inevitable: a scoping review of death and dying education in U.S. medical schools. Acad Med.

[16] Göktuna G, Dağcan Şahin N, Gürol Arslan G (2025). Death literacy and related factors among nursing students in Turkey: the role of spiritual well-being. J Relig Health.

[17] Vitorino J, Querido A, Semeão I, Laranjeira C (2024). Promoting death literacy in palliative care nursing education using narrative pedagogy. Proceedings of the 10th International Conference on Higher Education Advances.

[18] Chan WC, Tin AF (2012). Beyond knowledge and skills: self-competence in working with death, dying, and bereavement. Death Stud.

[19] Zhu JJ, Cui XS (2024). A study of death education for nursing students: scoping review. Nurs Commun.

[20] Corr CA, Corr DM, Bryant CD (2003). Death education. Handbook of Death and Dying.

[21] Dickinson GE, Mermann AC (1996). Death education in U.S. medical schools, 1975-1995. Acad Med.

[22] Schmit JM, Meyer LE, Duff JM, Dai Y, Zou F, Close JL (2016). Perspectives on death and dying: a study of resident comfort with end-of-life care. BMC Med Educ.

[23] Noonan K, Grindrod A, Shrestha S, Lee S, Leonard R, Johansson T (2024). Progressing the Death Literacy Index: the development of a revised version (DLI-R) and a short format (DLI-9). Palliat Care Soc Pract.

[24] (2023). National Statement on Ethical Conduct in Human Research 2023. National Health and Medical Research Council.

[25] Braun V, Clarke V (2008). Using thematic analysis in psychology. Qual Res Psychol.

[26] Galiana L, Oliver A, De Simone G, Linzitto JP, Benito E, Sansó N (2019). A brief measure for the assessment of competence in coping with death: the Coping with Death Scale short version. J Pain Symptom Manage.

